# Computational immunology in venom research: a systematic review of epitope prediction and validation approaches

**DOI:** 10.1093/bib/bbaf519

**Published:** 2025-10-03

**Authors:** Razana Zegrari, Abderrahim Ait Ouchaoui, Zainab Gaouzi, Hanane Abbou, Rihab Festali, Rachid Eljaoudi, Saber Boutayeb, Lahcen Belyamani, Ilhame Bourais

**Affiliations:** Mohammed VI University of Sciences and Health (UM6SS), Boulevard Mohammed taïeb Naciri, Commune Hay Hassani 82 403, 20100, Casablanca, Morocco; Mohammed VI Center for Research and Innovation (CM6RI), 01, Boulevard Mohamed Al Jazouli, Madinat Al Irfane, Hay Riad, 10112, Rabat, Morocco; Mohammed VI University of Sciences and Health (UM6SS), Boulevard Mohammed taïeb Naciri, Commune Hay Hassani 82 403, 20100, Casablanca, Morocco; Mohammed VI Center for Research and Innovation (CM6RI), 01, Boulevard Mohamed Al Jazouli, Madinat Al Irfane, Hay Riad, 10112, Rabat, Morocco; Mohammed VI University of Sciences and Health (UM6SS), Boulevard Mohammed taïeb Naciri, Commune Hay Hassani 82 403, 20100, Casablanca, Morocco; Mohammed VI Center for Research and Innovation (CM6RI), 01, Boulevard Mohamed Al Jazouli, Madinat Al Irfane, Hay Riad, 10112, Rabat, Morocco; Mohammed VI University of Sciences and Health (UM6SS), Boulevard Mohammed taïeb Naciri, Commune Hay Hassani 82 403, 20100, Casablanca, Morocco; Mohammed VI Center for Research and Innovation (CM6RI), 01, Boulevard Mohamed Al Jazouli, Madinat Al Irfane, Hay Riad, 10112, Rabat, Morocco; Mohammed VI University of Sciences and Health (UM6SS), Boulevard Mohammed taïeb Naciri, Commune Hay Hassani 82 403, 20100, Casablanca, Morocco; Mohammed VI Center for Research and Innovation (CM6RI), 01, Boulevard Mohamed Al Jazouli, Madinat Al Irfane, Hay Riad, 10112, Rabat, Morocco; Mohammed VI Center for Research and Innovation (CM6RI), 01, Boulevard Mohamed Al Jazouli, Madinat Al Irfane, Hay Riad, 10112, Rabat, Morocco; Biotechnology lab (MedBiotech), Bioinova Research Center, Medical and Pharmacy School, Mohammed V University Av. Mohamed Belarbi El Alaoui Rabat Institut, 10000, Rabat, Morocco; Mohammed VI University of Sciences and Health (UM6SS), Boulevard Mohammed taïeb Naciri, Commune Hay Hassani 82 403, 20100, Casablanca, Morocco; Mohammed VI Center for Research and Innovation (CM6RI), 01, Boulevard Mohamed Al Jazouli, Madinat Al Irfane, Hay Riad, 10112, Rabat, Morocco; Mohammed VI University of Sciences and Health (UM6SS), Boulevard Mohammed taïeb Naciri, Commune Hay Hassani 82 403, 20100, Casablanca, Morocco; Mohammed VI Center for Research and Innovation (CM6RI), 01, Boulevard Mohamed Al Jazouli, Madinat Al Irfane, Hay Riad, 10112, Rabat, Morocco; Department of Emergency, Mohammed V Military Training Hospital, Mohammed V University of Rabat, Av. Abderrahim Bouabid,10045, Rabat, Morocco; Mohammed VI University of Sciences and Health (UM6SS), Boulevard Mohammed taïeb Naciri, Commune Hay Hassani 82 403, 20100, Casablanca, Morocco; Mohammed VI Center for Research and Innovation (CM6RI), 01, Boulevard Mohamed Al Jazouli, Madinat Al Irfane, Hay Riad, 10112, Rabat, Morocco; Laboratory of Human Pathologies Biology, Faculty of Sciences, Mohammed V University, 4 Avenue Ibn Batouta, 1014, Rabat, Morocco

**Keywords:** immunoinformatics, epitope prediction, venom toxins, antivenom development, *in silico* validation, B-cell, T-cell, epitopes

## Abstract

Venom-based therapies are hindered by traditional discovery methods that are costly and inconsistent. Immunoinformatics offers a faster route to identify immunogenic epitopes, yet its application to venom proteins remains limited. We conducted a systematic review under PRISMA-2020 guidelines to identify studies predicting venom toxin epitopes computationally and validating them experimentally. Risk of bias was evaluated using a custom 20-question checklist. Following our systematic search, 11 articles met inclusion criteria. Multitool prediction strategies consistently outperformed single-tool approaches, particularly when structural and sequence-based models were combined. Experimental validations confirmed immunogenicity through diverse assays, but reporting inconsistencies, limited negative data, and variable study designs impaired direct comparison. Toxin family and structural data availability emerged as key factors influencing prediction success. *In silico* epitope prediction, combined with experimental validation, holds strong promise for advancing venom research. Our systematic bias assessment underscores the critical need for standardized frameworks to evaluate dataset selection, algorithm parameters, and validation rigor in computational epitope discovery. Moreover, the field must urgently address data scarcity, standardize validation protocols, and expand venom-specific training datasets to fully realize the promise of immunoinformatics-driven discovery.

## Introduction

Envenomation by venomous animals remains a significant global health challenge, particularly in tropical and subtropical regions where access to effective treatments is often limited [1, 2]. Venom research has traditionally focused on the development of antivenoms through animal immunization with crude venom, a method fraught with limitations such as batch variability, limited cross-reactivity, and the risk of adverse immune reactions [3]. However, the field is undergoing a transformative shift, driven by new-generation methods to harness *in silico* tools for the development of safe, cost-effective, and potent treatments against envenomation [4]. These computational approaches offer a venom-independent pathway to accelerate the discovery of next-generation therapeutics, diagnostics, and vaccines, addressing the urgent need for safer, more efficient, and broadly effective solutions [5, 6].

Computational biology offers a fast lane toward producing better treatments in a faster, more guided fashion. Where subjects were previously tested blindly in laboratories until serendipitous events led to discovery, bioinformatics provides more focused and conscious theories and laboratory candidates [7, 8]. Bioinformatics is a relatively recent discipline that still faces challenges such as reproducibility and false positives [9, 10]. Immunoinformatics is an especially recent field involving the development and application of computational algorithms to predict and map potential B-cell and T-cell epitopes. This approach enables the identification of antigenic regions within target proteins, offering valuable insights into immune recognition. High-performance immunoanalytic tools used for epitope prediction have broad applications, including the design of peptide-based vaccines, the diagnosis and monitoring of immune-related diseases, and the characterization of antibody responses. By supporting the accurate identification of immunologically relevant sites, immunoinformatics plays a central role in advancing our understanding of immune mechanisms and in guiding the development of targeted immunotherapies [11–13]. Qualitative research helps improve these new emerging fields positively and fine-tunes their application in an optimized manner, insuring scientific integrity and reproducibility [14].

In venom research, where the diversity and complexity of toxin profiles present significant challenges, immunoinformatics has emerged as a transformative tool. *In silico* methods now offer the ability to process vast venom protein datasets, identify conserved and immunogenic regions, and even predict cross-reactive epitopes that could contribute to broad-spectrum antivenom formulations [5, 15, 16]. This is particularly crucial in regions affected by multiple venomous species, where there is a pressing need for polyvalent and accessible therapies.

Central to this advancement is the mapping of B-cell and T-cell epitopes, short antigenic sequences recognized by the immune system, which enables the rational design of targeted interventions such as subunit vaccines or neutralizing antibodies [17]. While this process once depended entirely on time-consuming experimental methods, the integration of computational approaches has significantly accelerated and refined epitope discovery. Immunoinformatics tools can not only predict potential epitopes with speed and scalability but also reduce reliance on whole venom immunizations, improving both safety and efficiency in antivenom development [18–20].

As such, immunoinformatics is not merely supporting venom research, it is reshaping its methodology, offering a new framework where epitope prediction, cross-reactivity analysis, and therapeutic design are guided by data-driven, computational insights. This systematic review explores the emerging role of epitope mapping in venom research, highlighting its applications in antivenom development, diagnostics, and vaccination. By evaluating the evidence supporting this approach, we aim to underscore the rationale for integrating computational predictions with experimental validation, paving the way for a new era of both innovative and impactful venom research. Through this lens, we seek to address the need for efficient therapies and diagnostics, ultimately improving outcomes for millions affected by envenomation worldwide.

## Materials and methods

This systematic review is reported according to the PRISMA 2020 guidelines. We registered and published the protocol with PROSPERO (CRD420250640879). Two independent researchers conducted all phases of this systematic review, with any discrepancies resolved with a third reviewer.

### Search question

The search question steering this systematic review is: what are the immunogenic epitopes derived from venom toxins that were predicted using *in silico* methods and experimentally validated?

This systematic review investigates immunogenic epitopes from venom toxins that have been computationally predicted and experimentally confirmed. Following the P(E)Co framework, the problem (P) focuses on venom-derived immunogenic epitopes, the exposure (E) includes studies that employed *in silico* methods for epitope prediction, and the context (Co) is limited to research where these predicted epitopes were validated through laboratory experiments.

### Search strategy

A systematic search was conducted to identify research articles that utilized *in silico* methods to predict immunogenic epitopes derived from venom toxins and subsequently validate these predictions experimentally. The search strategy was designed to capture a comprehensive dataset of studies from multiple electronic databases, including Scopus, PubMed, PMC, and Google Scholar. Combinations of keywords, MESH terms, and Boolean operators were used, focusing on terms such as (“venom toxin” OR “snake venom” OR “scorpion venom” OR “bee venom” OR “arachnid venom” OR “spider venom”) AND (“epitope prediction” OR “epitope mapping” OR “B-cell epitope” OR “T-cell epitope” OR “*in silico* prediction” OR “computational prediction”) AND (“antivenom development” OR “antivenom production” OR “antivenin” OR “neutralizing antibodies” OR “immunotherapy” AND NOT [“virus” OR “viral” OR “bacteria” OR “bacterial” OR “pathogen”]).

No restrictions were placed on the publication year. All papers retrieved were in the English language. Manual searches were also performed to identify additional articles not retrieved through automated database queries.

### Inclusion and exclusion criteria

Studies were included in this review if they employed *in silico* methods to predict immunogenic epitopes from venom toxins and provided experimental validation of these predictions. Only articles that explicitly reported both computational predictions and laboratory-based validation were considered. Studies that tested epitopes that were experimentally identified rather than computationally predicted were excluded. Additionally, studies that relied solely on *in silico* validation methods, such as molecular dynamics simulations, without experimental confirmation, were not included. Duplicate records, including preprints of subsequently published articles, were removed, with the final peer-reviewed version retained. Non-English studies, review articles, and conference abstracts were also excluded.

### Data extraction and analysis

Two reviewers independently extracted data using a predefined form that was piloted and refined. The extracted information included study details (location, year, study design), computational approach (*in silico* prediction tools used), experimental validation methods, and key outcomes (epitope immunogenicity, antibody response, and neutralization capacity).

Extracted data were then organized into tables to compare *in silico* prediction tools, experimental validation methods, and key findings across studies. Studies were grouped based on the prediction methods and experimental approaches used, allowing for a structured comparison. A narrative synthesis was then conducted to identify methodological patterns, assess consistency in outcomes, and examine discrepancies between computational predictions and experimental validation.

Any discrepancies between reviewers were resolved by consensus, with input from a third reviewer when necessary.

### Assessment of the risk of bias

Due to the absence of standardized guidelines for evaluating bias in studies that integrate bioinformatics with experimental validation, a custom assessment approach has been adopted. The quality of computational modelling will be assessed using an adapted version of the Strengthening the Reporting of Empirical Simulation Studies (STRESS) checklist [21], ensuring methodological rigor in *in silico* analyses. Additionally, the risk of bias will be evaluated using a checklist derived from existing literature, including the framework by Medeiros *et al.* (2024) [22], which was developed for assessing both *in silico* and *in vivo* studies. The 20-item bias assessment checklist was created specifically for studies that combine *in silico* epitope prediction with experimental validation. The items were chosen to reflect core scientific principles that directly impact reproducibility and validity [21, 23]. Questions about study rationale, inclusion criteria, and reporting of parameters ensure research hypotheses are transparent and methods are reproducible. Items related to data handling, software versions, and tool parameters address computational reproducibility, which is vital in *in silico* studies. Likewise, questions on ligand selection, structural validation, and docking parameters identify potential sources of modeling bias. For experimental validation, items concerning controls, replication, animal randomisation, and ethical statements align with widely accepted standards in preclinical research, ensuring internal validity and humane practices. Finally, questions regarding reporting negative results, availability of supplementary materials, and outcome transparency help prevent selective reporting bias. Each item was scored equally (1 = reported, 0 = absent, 0.5 = partial compliance); “NA” items were excluded from the total, ensuring studies weren’t penalized for methodological aspects irrelevant to their design. This checklist offers a thorough framework to assess bias risk in translational studies combining computational predictions with experimental validation.

## Results

**Figure 1:**
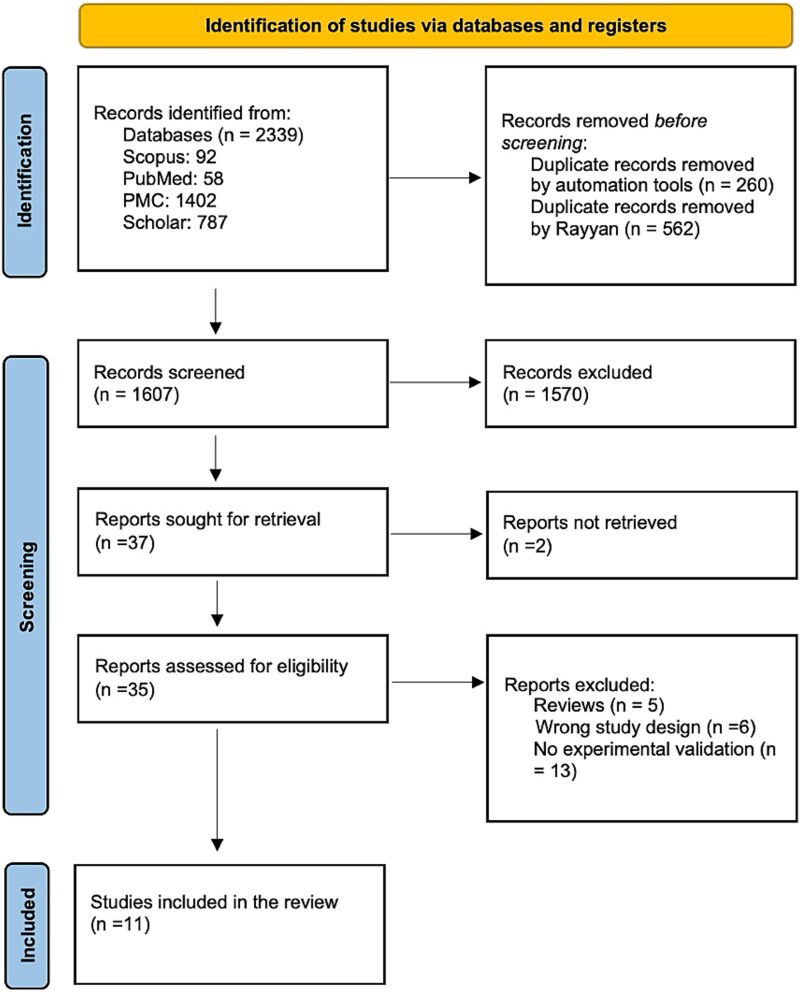
PRISMA chart illustrating the study selection and screening process.

The systematic search and selection process followed the PRISMA 2020 guidelines, as illustrated in the flow diagram (Fig. 1). A total of 2339 records were initially identified across five major databases: PubMed (58 records), Scopus (92 records), PMC (1402 records), Google Scholar (787 records), and additional sources. After the removal of duplicates (*n* = 822) using automated tools and manual curation through Rayyan, 1607 unique records were screened for relevance based on title and abstract. During this screening stage, 1570 records were excluded primarily because they did not focus on venoms or venom-derived proteins. The majority of these records addressed unrelated fields such as viral epitopes, bacterial antigens, allergens, vaccine development for nonvenom pathogens, or general immunoinformatics without toxin relevance. Additional reasons for exclusion included studies that lacked computational epitope prediction, studies limited to molecular docking or structural modeling without immunogenicity assessment, and records focusing solely on therapeutic antibodies without epitope identification. Full-text retrieval was sought for 37 reports, of which two could not be accessed. Of the 35 full-text articles assessed for eligibility, 24 were excluded for reasons such as being reviews (*n* = 5), using an inappropriate study design (*n* = 6), or lacking experimental validation (*n* = 13) (Table S1). Ultimately, 11 studies met the inclusion criteria and were included in the final review. These studies were selected based on their use of *in silico* methods to predict venom toxin-derived immunogenic epitopes and their subsequent experimental validation. The following sections present detailed findings from these studies, focusing on the methodologies employed, the specific venom-derived epitopes identified, and their potential therapeutic applications.

### Overview of extracted articles

The reviewed studies (Table 1) highlight a global interest in venom immunogenicity, with research spanning multiple continents. Snake venoms dominate the dataset, with metalloproteinases extensively analyzed. In South America, *Bothrops atrox, B. asper*, and B*. leucurus* from Brazil were studied by Kozlova *et al.* 2018 [24], while Wagstaff *et al.* 2006 [20] examined *Echis ocellatus* metalloproteinases in Africa. Research in Asia focused on *Deinagkistrodon acutus* (Cao *et al.* 2016 [25]), *Sinonatrix annularis* (Li and Xiong *et al.* 2017 [26]), and *Trimeresurus stejnegeri* alongside *Protobothrops mucrosquamatus* (Long *et al.* 2021 [27]). The cytotoxins of *Naja* cobras in Asia were also investigated by Hiu *et al.* 2023 [28]. Beyond metalloproteinases, serine proteases were examined in *Bothrops* and *Lachesis* species from South and Central America (Madrigal *et al.* 2017 [29]). Phospholipases, particularly PLA2, were studied in snake venoms from Asia and South America (Cao *et al.* 2016; Li and Xiong *et al.* 2017). Scorpion venom research included *Androctonus australis* and *Allopauropus mauretanicus mauretanicus* from Africa (Alvarenga *et al.* 2010 [30]). Spider venom studies focused on species from North and South America, with *Latrodectus* from North America (Davydov *et al.* 2009 [31]) and *Loxosceles intermedia*, L. laeta, and *L. gaucho* from South America (De Moura *et al.* 2011 [32]). Additionally, Machado-de-Ávila *et al.*, 2014 [33] investigated Mutalysin-II from *Lachesis muta muta* in South America. These findings illustrate the geographical diversity of venom research and the range of toxins investigated across different regions.

**Table 1:** General information on selected studies

Year	study	Geographical region	Peptide class/name	Species
2009	Davydov *et al.* 2009	North America	α-Latrotoxin	Spider: *Latrodectus mactans*
2017	Madrigal *et al.* 2017	South and central America	Venom serine proteinases (SVSPs)	Viperidae: *L. stenophrys*
2011	De Moura *et al.* 2011	South America	Dermonecrotic factor (DNF) family	Spiders: *L. intermedia (Li)*, *L. laeta* and *L. gaucho*
2014	Machado-de-Ávila *et al.* 2014	South America	Mutalysin-II	Snake: *L. muta muta*
2021	Long *et al.* 2021	Asia	Phospholipase A2 and snake venom metalloproteinase TM-3	Snakes: *T. stejnegeri* and *P. mucrosquamatus*
2006	Wagstaff *et al.*2006	Africa	Snake venom metalloproteinases (SVMPs)	Snake: *E. ocellatus*
2021	Hiu *et al.* 2023	Asia	Cytotoxin	Snake: *Naja*
2017	Li and Xiong *et al.* 2017	Asia	Phospholipase A2	Snake: *S. annularis*
2018	Kozlova *et al.* 2018	South America	Atroxlysin-I	Snake: *B. atrox*, *B. asper*, and *B. leucurus*
2010	Alvarenga *et al.* 2010	Africa	Amm VIII	Scorpions: *A. australis* and *A. mauretanicus*
2016	Cao *et al.* 2016	Asia	Serine protease, metalloprotease, and phospholipase A2	Snake: *Deinagkistrodon (D) acutus*

### Bias risk assessment

**Figure 2:**
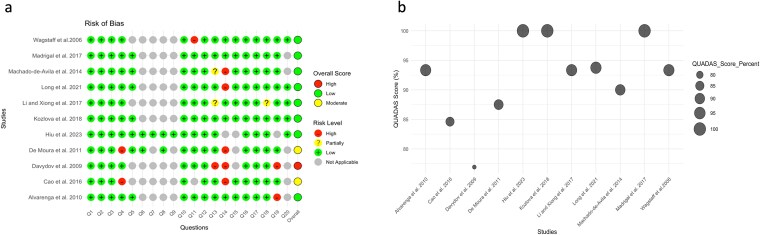
Risk of bias assessment of selected studies. Panel A: Dot plot (traffic light plot) of domain-level evaluation across 20 questions, with green (low risk), yellow (partial risk), red (high risk), and grey (not applicable). Panel B: Bubble plot of overall QUADAS scores, with bubble size proportional to the percentage of low-risk responses. Studies with ≥90% low-risk responses were classified as low risk; 80%–89% as moderate risk; and <80% as high risk.

The 20-item appraisal tool was structured into four domains: general rationale (Q1–Q3), in silico reporting (Q4–Q11), experimental design (Q12–Q18), and article-wide good-practice items (Q19–Q20) (Fig. 2, Table S2). All 11 papers satisfied the first domain by providing a coherent biological premise, describing data-filtering decisions, and specifying inclusion criteria. Divergence emerged in the *in silico* block. Wagstaff *et al.* 2006; Davydov *et al.* 2009; Kozlova *et al.* 2018; Long *et al.* 2021 detailed software versions, tool parameters and three-dimensional inspection procedures, and they also reported epitopes that failed downstream validation. By contrast, Machado-de-Ávila *et al.* 2014, Li & Xiong *et al.* 2017 and Alvarenga *et al.* 2010 omitted operating-system or build information and disclosed only successful predictions, leaving Q9 and Q11 unmet. The widest variability lay in experimental reporting. While positive and negative controls were common (e.g. Madrigal *et al.* 2017; Cao *et al.* 2016), explicit biological replication and animal randomisation were documented in only four studies, most comprehensively by Kozlova *et al.* 2018 and Long *et al.* 2021, and were entirely absent in Li & Xiong *et al.* 2017 and Alvarenga *et al.* 2010. Drop-out handling and blinding were seldom addressed. Finally, eight of nine animal studies included an ethics statement, yet only six articles, led by Davydov *et al.* 2009 and Wagstaff *et al.* 2006, made raw data or scripts publicly available. Collectively, the corpus exhibits strong conceptual framing, acceptable but sometimes incomplete computational transparency, and uneven experimental rigour, underscoring a need for consistent reporting of software environments, negative results, replication strategies and data-sharing to elevate all contributions to the standard set by the best-documented papers.

### Review of themes and findings

A total of 11 studies met the inclusion criteria, all of which employed immunoinformatics-based epitope prediction followed by experimental validation to assess the immunogenicity of venom-derived epitopes. The selected studies varied in computational approaches, experimental models, and validation techniques, highlighting the diverse methodologies used to bridge *in silico* predictions with biological confirmation.

### Venom research and epitope prediction: diverse aims and applications

Venom research has increasingly leveraged *in silico* epitope prediction to identify immunogenic regions within venom toxins, aiming to develop targeted therapeutic and diagnostic tools. The reviewed studies demonstrate various aims, reflecting the diverse applications of venom-derived epitopes in biomedical research.

The majority of studies focused on antivenom development, aiming to neutralize the toxic effects of venom through the identification of epitopes that elicit protective immune responses. For example, Davydov *et al.* (2009) predicted and validated epitopes from *Latrodectus* spider venom, demonstrating the potential for antivenom targeting spider envenomation. Similarly, Machado-de-Ávila *et al.* (2014) identified epitopes from *L. muta muta* venom that protected against hemorrhagic effects, highlighting the therapeutic potential of venom-derived antibodies. Li and Xiong *et al.* (2017) also contributed to this goal by producing monoclonal antibodies against *S. annularis* venom, further underscoring the importance of epitope prediction in antivenom development.

Several studies advanced the concept of cross-neutralization, where antibodies generated against epitopes from one venom species demonstrated reactivity against heterologous venoms. For example, Madrigal *et al.* (2017) identified peptides that elicited antibodies with cross-reactivity to *Bothrops* and related snake venoms, suggesting the potential for broad-spectrum antivenoms. Similarly, Wagstaff *et al.* (2006) designed a synthetic DNA immunization construct (EoSVMP string) that induced cross-generic and cross-specific antibody responses, effectively neutralizing venom from multiple snake species. These findings highlight the potential for developing antivenoms that can target multiple venomous species, reducing the need for species-specific treatments.

Beyond antivenom development, some studies explored the use of venom-derived epitopes for diagnostic tools. For instance, Long *et al.* (2021) developed diagnostic antibodies for *Trimeresurus* and *Protobothrops* genera, enabling the detection of venom components in clinical samples. These diagnostic tools are critical for rapid and accurate identification of envenomation, particularly in regions where multiple venomous species coexist. The ability to quickly identify the source of envenomation can significantly improve treatment outcomes and reduce morbidity.

Additionally, alternative immunization strategies were investigated, such as peptide-based vaccines and DNA immunization. Kozlova *et al.* (2018) demonstrated that computationally predicted epitopes from *Bothrops* snake venom could generate protective immunity in animal models, highlighting the potential for peptide-based vaccines. Wagstaff *et al.* (2006) further advanced this field by using DNA immunization to induce neutralizing antibodies against venom metalloproteinases, showcasing the versatility of immunization strategies in venom research. These innovative approaches could potentially reduce the reliance on traditional antivenoms and provide long-term protection against venomous bites.

### Computational approaches in epitope prediction

The studies included in this review employed a range of *in silico* methodologies for epitope prediction, broadly categorized into AI-driven models, structure-based matrix approaches, and hybrid or sequence-based tools (Table 2). Sequence-based and machine-learning approaches were utilized by Cao *et al.* (2016) through DNAStar, IEDB, and BepiPred Linear Epitope Prediction, while Davydov *et al.* (2009) integrated a basic vector method with support vector machines (SVM). Structure-based strategies were applied by De Moura *et al.* (2011) and Machado-de-Ávila *et al.* (2014), who used MIMOP and Blue Star STING (BSS), respectively, to predict conformational epitopes. Traditional antigenic peptide prediction methods were employed by Madrigal *et al.* (2017) and Li *et al.* (2017) using the ABCPred server and the DNAStar Protean program. Kozlova *et al.* (2018) implemented a decision tree classifier enhanced by the SMOTE algorithm. Hiu *et al.* (2023) adopted an integrated framework combining NetCTL, BepiPred-2.0, and the IEDB Analysis Resource, along with ABCPred, Ellipro, and classical physicochemical methods including Karplus & Schulz Flexibility, Kolaskar & Tongaonkar antigenicity, and Parker hydrophilicity predictions. Additional studies such as Long *et al.* (2021) applied the Protean (Lasergene) platform, Wagstaff *et al.* (2006) utilized Jameson-Wolf antigenic profiling, and Alvarenga *et al.* (2010) employed PEPOP. The diversity of these computational approaches illustrates the ongoing evolution of immunoinformatics and underscores the complementary nature of different predictive strategies in advancing venom epitope identification.

**Table 2:** Summary of toxins, epitope prediction tools, and selected epitopes in reviewed studies

Study name	Name of toxin	Type of tool used	Name of tool	Epitopes selected
Cao *et al.* (2016)	Serine Protease (SP), metalloprotease (MP), phospholipase A2 (PLA2)	Sequence-based antigenicity prediction and AI-Based (Random Forest)	DNAStar, IEDB, BepiPred Linear epitope prediction	SE1 (IQFDDEQR-RYAI), SE2 (IDTCNQDSGGPL), MET1 (TALPKGAVQQKY), MET2 (DYSETHYSPDGR), PL1 (MQEMCECD-KAFA), PL2 (DCDSKKDRYSYK)
Davydov *et al.* (2009)	Latrophilin 1, 2, and 3	Statistical modeling	Basic vector method, support vector machine (SVM)	CYAFNTNANREEPVSLAFPNP (Latrophilin 1), CQRGPVSSTVAGPQEGSRGTK (Latrophilin 2), CSTTPSLPGRRNRSTSTPSPA (Latrophilin 3), CHDTSPYRWGGKTDIDLAVDE (Common)
De Moura *et al.* (2011)	LiD1 (Sphingomyelinase D)	AI-based multiple instance learning (MIL)	MIMOP	NCNKNDHLFACW, ECKSDWMPPYCP, ECTQKYDWLFCM, QDEERVSSCPKVAWTFC, PER: C197, Y224, W225, T226, D228, K229, R230, T232, Y248
Machado-de-Ávila *et al.* (2014)	Mutalysin-II (Mut-II)	Structure-based prediction	Blue Star STING (BSS)	Discontinuous Epitope, 25 residues selected: K19, N21, N25, N36, N59, Q60, N64, N70, K74, N86, S89, N104, Y116, P117, K149, N153, S158, P172, Q185, M186, K190, K192, Q194, L197, K199
Madrigal *et al.* (2017)	Serine proteinase (Q072L7)	AI-Based (artificial neural network)	ABCPred server	(YAQKSSELVIGCDECN1), (LVYITSCHCCCTLIN), (WWITAAHCDR), (NMLIFFDVHSJK), (RVAKERFCPNRKKDD), S6 (KINOMILIKLDSPVSNS), (EHLAPLSIPSNPSVGSVCRI), (GWCAITSPWTLPCVPH), (ANINILDYPEVCREATT), (TCLPATSRTLCAGILE), (LEGGKDSGKGDSGGPLI), (QGVSWCAHPCCQSLRP), (YTEWIQSLACNADATC).
Li *et al.* (2017)	PLIγ (SaPLIγ)	Sequence-based prediction	DNAStar Protean program	CPVLRLSNRTHEANRNDLIKVA
Kozlova *et al.* (2018)	Atroxlysin-I (Atr-I), metalloendopeptidase (Bap1) and Leucurolysin-a (Leuc-a)	Sequence-based prediction	Decision tree classifier	Eight epitopes for Atr-I: QQR, FIVVDHGMF, DKIRRRIH, FGEWR, IQDHSEQDLM, HDTG,CIMS, SDCS. Six for Bap1: VVADHG, NTVGF, DVHA, KSFGEWRERD, GAKSCIMAS, SYEPSDCSQNQYE. Six for Leuc-a: VVADHGMFKKYN, NTVNGFFRSMN, FGEWRER, AGMCDLSQSVAVVMDHS, NLGMRHDGNQCHCNAPSCIMAD, FEPSDCSQ,
Long *et al.* 2021	Phospholipase A₂ (PLA₂) and snake venom metalloproteinase	Sequence-based statistical method	Protean (Lasergene)	PLA2: QETGKNPATSYG, INLKLFCKKTSEQ, SVMP: TKYSSNFKKI, QADAPTTAG, EHDDKDKCK
Wagstaff *et al.* (2006)	Snake venom metalloproteinase (SVMP)	Sequence-based statistical method	Jameson-Wolf antigenic profiling	EoMPep 1 (zinc-binding motif), EoMPep 2 (RGD motif), EoMPep 3a/3b (GEECDC box), EoMPep 4 (SECD/DCD motif), EoMPep 5 (VKC-like motif), EoMPep 6 (C-terminal region)
Alvarenga *et al.* (2010)	Amm VIII (scorpion toxin) and AaH II (scorpion toxin)	3D structure-based epitope mapping	PEPOP	24 discontinuous-continuous peptide sequences: OWAPYCYERNAYNEIKLK, RWAYNEIKLESYKLKD, YFOWAPYCYYYNEIKLK, PYCOWAYYYNDNKEKK, NUNRTKYOWAPYCYF, NUNRTKYYYNEIKLK, IKLKESKEYYYEYDWA, PYCOWAYYYERNAYNE, DHRTKYYYNEIKLKES, RTKYYYNEIKLESYK, YKILKOVYYNEIKLKES, YVOWAPYCYERNAYNE, YYYNEIKLESYKLKD, ESNEIKLYKILKOVY, DHLRYKSENEIKLK, LKOVYESENEIKLKDH, NEIKLKESYKLKOVY, PGKNOPYFOWAPYCY, YNEIKLKESYKLKOV, RWAYNEIKLESYK, IKLKESKEYRNAY, LKOVKOH, DHLRYNK, YKILKOH,
Hiu *et al.* (2023)	Cobra venom cytotoxin (CTX)	AI-based (SVM),	NetCTL,	KLVPLFY, CPAGKNLCY, MFMVSTPTKVP, DVCPKNSLL,.
		AI (Random Forest)	BepiPred-2.0,	
		Hybrid model	IEDB analysis resource,	
		AI-Based (artificial neural network)	Antigenic peptide prediction method,	
		Artificial neural network–ANN	ABCPred	
		Structure-based	Ellipro	
		Statistical modeling (mobility-based algorithm)	Karplus & Schulz flexibility prediction	
		Statistical (physicochemical-based algorithm)	Kolaskar & Tongaonkar antigenicity prediction–	
		Statistical (hydrophobicity-based algorithm)	Parker hydrophilicity prediction	

### Experimental methods for validation of *in silico* predictions

The experimental validation of *in silico* predictions in the reviewed studies can be divided into two categories: (i) those using single or limited techniques and (ii) those employing multiple techniques for comprehensive validation (Table 3).

**Table 3:** Summary of experimental methods and confirmed epitopes in reviewed studies

Study	Experimental method	Confirmed epitope	Number of predicted epitopes	Number of confirmed epitopes
Cao *et al.* (2016)	- ELISA: measured antibody titers. - *In vivo* neutralization assay: tested antivenom efficacy.	SE1 (IQFDDEQR-RYAI), SE2 (IDTCNQDSGGPL), MET1 (TALPKGAVQQKY), MET2 (DYSETHYSPDGR), PL1 (MQEMCECD-KAFA), PL2 (DCDSKKDRYSYK)	Six	Six
Davydov *et al.* (2009)	- Immunostaining: confirmed antibody binding. - Immunoblotting: verified antibody specificity.	CYAFNTNANREEPVSLAFPNP (Latrophilin 1), CQRGPVSSTVAGPQEGSRGTK (Latrophilin 2), CSTTPSLPGRRNRSTSTPSPA (Latrophilin 3), CHDTSPYRWGGKTDIDLAVDE (Common)	Four	Two
De Moura *et al.* (2011)	- Indirect ELISA: assessed antibody reactivity. - *In vivo* neutralization assay: tested protection against venom effects.	NCNKNDHLFACW, NSNKNDHLFASW, PER: C197, Y224, W225, T226, D228, K229, R230, T232, Y248	Four mimotopes discontinuous epitopes	Two mimotopes, nine residues were predicted as the potential epitopic region
Hiu *et al.* (2023)	- Site-directed mutagenesis: validated epitope locations. - Cytotoxicity assay: tested cytotoxicity of mutants.	KLVPLFYK, AGKNL, “MFMVSTPKVPV” and “DVCPKNSLL”	Four epitopes	Four linear epitopes common between B- and T- cells
Machado-de-Ávila *et al.* (2014)	- ELISA: to test antibody binding to Mutalysin-II. - Western Blot: to confirm antibody recognition of Mutalysin-II. - Hemorrhagic assay: to test the neutralizing ability of antibodies.	Final peptide of 11 amino acids P117-Y116-C115-Q194-C195-L197-N198-K199-P200-Y5-L48 confirmed as epitope.	Discontinuous epitope: 25 amino acid residues	11 amino acids confirmed
Madrigal *et al.* (2017)	- Capture ELISA: to test immunoreactivity of synthetic peptides against antivenoms. - SPOT Synthesis: to map epitopes using cellulose-bound peptides.	S3: WVLTAAHCDR, S6: KDKDIMLIKLDSPVSNS and S11: LEGGKDSCKGDSGGPLI	13	three
Li *et al.* (2017)	- Indirect ELISA: to test antibody binding to synthetic peptides. - Western Blot: to confirm antibody recognition of recombinant and natural PLIγ. - Immunization of mice: to produce monoclonal antibodies against PLIγ.	CPVLRLSNRTHEANRNDLIKVA	one	one
Kozlova *et al.* (2018)	- SPOT Immunoblotting: to map epitopes in Atr-I, Bap1, and Leuc-a. - ELISA: to monitor antibody production against synthetic peptides - *In vitro* neutralization Assay: to test neutralizing activity of antibodies. - *In vivo* hemorrhagic Assay: to test neutralization of Atr-I-induced hemorrhage.	Atr-I: two epitopes (aa 19–39 and 46–75), Bap1: two epitopes, Leuc-a: three epitopes. AtrCPEN (aa 11–19) confirmed as neutralizing epitope	Eight epitopes for Atr-I six for Bap1 six for Leuc-a	Atr-I: two epitopes, Bap1: two epitopes, Leuc-a: three epitopes. AtrCPEN (computationally positive experimentally negative) confirmed as neutralizing epitope
Long *et al.* 2021	- ELISA: double-antibody sandwich ELISA to detect venom proteins. - Western Blot: to verify antibody specificity. - Peptide Synthesis: design and synthesis of genus-specific peptides. - Immunization: rabbits and mice immunized with peptide and protein antigens.	PLA2-pep-1, PLA2-pep-3 and SVMP-pep-1	Five	Three
Wagstaff *et al.* (2006)	- DNA Immunization: synthetic multiepitope - DNA immunogens were designed and used to immunize mice. - Immunoblotting: used to detect antibody reactivity against venom proteins. -ELISA: measured antibody titers against venom components. -*In vivo* neutralization assays: assessed the ability of antisera to neutralize venom-induced hemorrhage in mice	All seven Epitopes EoMPep 1–6	Seven	Seven
Alvarenga *et al.* (2010)	-Spot peptide synthesis: overlapping 12-mer peptides were synthesized using the Spot technique to map continuous epitopes. -Peptide immunoassay: antibody binding to peptides was detected using peroxidase-conjugated anti-rabbit IgG and chemiluminescence. -*In vivo* neutralization assays: mice were immunized with KLH-coupled peptides and challenged with a lethal dose of AaH II toxin to assess neutralization.	Seven discontinuous-continuous peptides: Peptide 2: (RWAYNEIKLESYKLKD), Peptide 3:(YFQWAPYCYYYNEIKLK), Peptide 4: (PYCQWAPYYYNDNKEKK), Peptide 6: (NUNRTKYYYNEIKLK), Peptide 13: (YYYNEIKLESYKLKD), Peptide 15: (DHLYKDYKESNEIKLK). And seven discontinuous-continuous peptides Cross-Reactive Peptides: Peptide 4 (PYCQWAPYYYNDNKEKK), Peptide 5 (NUNRTKYQWAPYCYF), Peptide 7 (IKLKESKEYYYEYDWA), Peptide 11 (YKILKQVYYNEIKLKES), Peptide 12 (YYYNEIKLESYKLKD), Peptide 15 (DHLYKDYKESNEIKLK), Peptide 20 (RWAYNEIKLESYK)	24	14

Several studies relied on single or limited experimental techniques to validate their predictions. For example, Davydov *et al.* (2009) focused on antibody production by conjugating peptides to KLH, immunizing rabbits, and validating through immunostaining and Western blotting (immunoblotting). Similarly, Madrigal *et al.* (2017) primarily used capture ELISA to assess antibody binding to synthetic peptides, without additional functional assays. Long *et al.* (2021) combined ELISA, Western blot, peptide synthesis, and immunization (in rabbits and mice), followed by simulation of envenomation both *in vitro* and *in vivo*, providing moderate functional validation. Wagstaff *et al.* (2006) employed DNA immunization, ELISA, immunoblotting, and *in vivo* neutralization assays to assess antibody binding and neutralization. Cao *et al.* (2016) utilized recombinant antigen production, ELISA, and *in vivo* neutralization assays to evaluate immunogenicity and functional neutralization.

While these studies provided important data on antibody recognition and neutralization, their reliance on a limited range of techniques may restrict the comprehensive validation of epitope structure and function.

In contrast, several studies implemented multiple complementary techniques for more robust validation. De Moura *et al.* (2011) combined SPOT synthesis, indirect ELISA, *in vivo* neutralization assays, and bioinformatic modeling to validate structural and functional properties of predicted epitopes. Machado-de-Ávila *et al.* (2014) employed peptide synthesis (Fmoc chemistry), liposome encapsulation, rabbit immunization, ELISA, Western blot, and hemorrhagic neutralization assays to verify both immunogenicity and functional efficacy. Li *et al.* (2017) combined peptide synthesis, mouse immunization, ELISA, and Western blotting to validate antibody production against synthetic peptides and natural/recombinant proteins. Kozlova *et al.* (2018) used a combination of SPOT immunoblotting, synthetic peptide immunization, ELISA, and *in vitro*/*in vivo* neutralization assays, supported by computational epitope prediction models, providing both functional and structural validation. Alvarenga *et al.* (2010) employed SPOT peptide synthesis, peptide immunoassays, ELISA, *in vivo* neutralization assays, and hydrophobicity analyses for thorough structural and functional confirmation of epitopes. Finally, Hiu *et al.* (2023) used advanced integrated methods, including MELD/LC–MS (epitope-omics), immunoturbidimetric assays, cell-free protein synthesis, site-directed mutagenesis, western blotting, cytotoxicity assays, and homology modeling, providing the most comprehensive multidimensional epitope validation among the reviewed studies.

### Validation rate of predicted epitopes

The reviewed studies demonstrated substantial variability in both epitope prediction and experimental validation success rates (Table 3). For instance, Cao *et al.* (2016) predicted six B-cell linear epitopes, all experimentally confirmed, indicating a high concordance between computational predictions and laboratory validation. In contrast, Davydov *et al.* (2009) predicted four epitopes for α-latrotoxin, with only two confirmed through immunostaining and immunoblotting, reflecting a moderate validation rate for machine-learning-based approaches.

De Moura *et al.* (2011), investigating spider venom, predicted four mimotopes along with a discontinuous potential epitopic region composed of nine residues. Two mimotopes were experimentally confirmed, alongside identification of key residues within the predicted region, suggesting partial validation success. Similarly, Machado-de-Ávila *et al.* (2014) identified a 25-amino acid discontinuous epitope from Mutalysin-II, with experimental assays confirming 11 amino acids as critical for immunogenicity and functional neutralization. Studies targeting snake venom serine proteases and phospholipases reported lower confirmation rates; Madrigal *et al.* (2017) confirmed three out of 13 predicted epitopes through immunoassays, while Li *et al.* (2017) validated one epitope using peptide immunization and ELISA, highlighting the challenges of epitope mapping in these toxin classes. Kozlova *et al.* (2018) predicted 20 epitopes across three metalloproteinases (Atr-I, Bap1, and Leuc-a) and experimentally confirmed seven epitopes, reflecting variability in prediction outcomes across different toxin families. Further, Long *et al.* (2021) predicted five and six epitopes using a combination of data mining and experimental validation, of which three were confirmed. Wagstaff *et al.* (2006) achieved full concordance, with all seven predicted epitopes experimentally validated following DNA immunization and functional assays. Alvarenga *et al.* (2010) predicted 24 epitopes, with 14 (plus seven cross-reactive peptides) confirmed, emphasizing the complexity of mapping both continuous and discontinuous epitopes. Finally, Hiu *et al.* (2023) predicted and confirmed four functional epitopes in cobra cytotoxins using advanced epitope-omics and mutagenesis validation strategies.

These results collectively display a wide range of prediction and validation efficiencies across different studies and toxin families.

## Discussion

The studies included in this review exhibit significant diversity in their approaches, spanning different venomous species, computational tools, and experimental validation techniques. The methodologies used for epitope prediction vary widely, ranging from AI-driven machine learning models to structural-based antigenicity assessments and sequence-based physicochemical analyses. Additionally, the experimental strategies for validating predicted epitopes differ considerably, including ELISA, Western blot, neutralization assays, and *in vivo* immunization studies. This variation in both computational and laboratory methods makes direct comparisons challenging, as each study applies distinct criteria for epitope selection, prediction, and confirmation. Despite these differences, common patterns emerge, particularly in the effectiveness of multitool validation approaches and the influence of toxin type on prediction success.

The reviewed studies demonstrate that *in silico* epitope prediction has become a cornerstone of modern venom research, particularly when combined with experimental validation. AI-driven tools, such as ABCpred and machine learning models, consistently identified linear B-cell epitopes that were later confirmed through immunoassays. Structural-based tools like PEPOP and Blue Star Sting proved particularly effective for toxins with resolved 3D structures, successfully predicting conformational epitopes involved in toxin neutralization. Hybrid approaches that integrate multiple prediction methods (e.g. MIMOP’s combination of sequence alignment and structural modeling) emerged as particularly promising, as they identified epitopes capable of eliciting cross-reactive antibodies. However, the field would benefit from more standardized validation protocols, as the depth of experimental confirmation varied significantly across studies, ranging from simple binding assays to comprehensive neutralization testing in animal models.

The divergence in methodologies among studies makes it difficult to quantify a precise success rate for epitope prediction tools. Variability in computational approaches from machine learning algorithms to structural-based models combined with a small pool of studies and limited data sets, prevents the establishment of a uniform metric for accuracy (a visualization is available in Fig. S3). However, studies that cross-correlated results using multiple tools, such as Hiu *et al.* (2023), demonstrated improved reliability in identifying immunogenic epitopes. This suggests that integrating diverse computational methods can enhance predictive performance, even though the variability in techniques and data quality precludes a precise trend analysis of tool accuracy.

Additionally, the tools that utilize artificial intelligence such as The Immune Epitope Database (IEDB) and its associated epitope prediction tools are trained on a comprehensive collection of experimentally validated epitope data derived from a wide array of sources. This includes epitopes related to infectious diseases, autoimmune disorders, allergies, and transplant rejection [34], If venom toxin epitopes are underrepresented or absent in these training datasets, the predictive models may not perform optimally for venom-related proteins. This limitation can increase the likelihood of false positives or less reliable predictions when analysing venom toxins. Therefore, while these tools are valuable for general epitope prediction, their accuracy for venom toxins may be compromised due to the lack of specific training data. Moreover, thorough validation is still needed when using prediction tools, as independent benchmarking of these tools for performance evaluation is to use and handle outputs of such tools carefully [35].

A strong trend emerged toward using predicted epitopes for antivenom development, with multiple studies demonstrating partial or complete neutralization of venom effects in preclinical models. Notably, research on snake venoms significantly outpaced studies on other venomous animals, despite the medical importance of spiders and scorpions in many regions [36]. While some investigations reported antibodies with cross-neutralizing potential, particularly against closely related species, truly broad-spectrum solutions remain elusive. The more limited but equally important work on diagnostic epitopes and vaccine candidates suggests these applications may represent underexplored opportunities in the field [37].

A major challenge identified in this review is the lack of detailed reporting on computational tool parameters, which affects the reproducibility of predictions. Many studies do not specify key settings such as threshold values, or algorithm modifications, making it difficult to assess how computational choices influence prediction outcomes. Additionally, negative results are often underreported, with studies primarily focusing on successfully validated epitopes while omitting cases where predicted epitopes failed experimental confirmation. This selective reporting can lead to an overestimation of the accuracy of epitope prediction tools. Furthermore, experimental validation methods vary significantly across studies, with differences in antigen preparation, antibody generation, and assay conditions, all of which impact reproducibility.

One of the key challenges in bioinformatics-based research is the absence of standardized guidelines for assessing risk of bias in computational studies. Unlike experimental research, which benefits from established frameworks for evaluating study quality, bioinformatics studies often lack clear criteria for assessing methodological rigor. Factors such as dataset selection, parameter tuning, and algorithm training can introduce bias, yet many studies do not provide sufficient details on these aspects. Developing standardized risk of bias assessment tools tailored for bioinformatics research would improve the reliability, reproducibility, and comparability of computational epitope prediction methods [21, 38].

Beyond the experimentally validated studies, several investigations relied exclusively on *in silico* pipelines, offering useful insights into the broader methodological landscape. Sequence-based predictors such as BepiPred, MHC2Pred, and SVM classifiers were frequently combined with population coverage or antigenicity profiling, while structure-based pipelines applied SwissModel, PEP-FOLD, and AutoDock Vina to model toxin–HLA interactions and generate candidate epitopes [18, 19, 39, 40]. Other hybrid approaches integrated conservation analysis, allergenicity prediction, or physicochemical profiling across diverse species, from snakes and spiders to jellyfish and honeybee allergen [18, 40–42]. Although these studies were excluded from the main analysis due to the lack of experimental validation, they nonetheless contribute preliminary epitope maps and innovative workflows. Their findings emphasize the value of computational exploration but also reinforce the need for standardized reporting and robust validation to transform predictions into meaningful biological and therapeutic outcomes.

Immunoinformatics has the potential to revolutionize the field of applied immunology, specifically in preventing disease through vaccine development or perfecting existing treatments for safer more effective care. In the case of antivenoms, traditional production methods being costly and demanding, give away a product with questionable reliability, adding to their cold chain dependency, and making their storage and distribution in rural areas, where they are needed the most, extremely challenging [5, 43, 44]. While antivenom development remains the primary focus, the exploration of cross-neutralization, diagnostic tools, and alternative immunization strategies highlights the broader impact of venom research in improving global health outcomes [45–48]. These diverse applications underscore the importance of continued innovation in epitope prediction and experimental validation to advance the field of venom immunology. Importantly, while *in silico* prediction combined with experimental validation shows promising potential, none of the reviewed studies have yet produced clinically available antivenoms. Most remain at proof-of-concept or preclinical stages, with validation limited to *in vitro* assays and animal models. Licensed antivenoms still rely on whole-venom immunization, reflecting the regulatory and technical hurdles of translating epitope-based strategies. Nevertheless, these studies lay essential groundwork for next-generation recombinant or epitope-focused antivenoms, underscoring the need for continued innovation in prediction and validation approaches.

## Conclusion

This systematic review highlights the considerable progress and ongoing challenges in the field of venom epitope prediction. *In silico* approaches, when combined with experimental validation, have demonstrated significant potential to accelerate the identification of immunologically relevant epitopes across diverse venom protein families. Our findings show that while some studies achieved high concordance between predicted and experimentally confirmed epitopes, others revealed variable success rates, often influenced by differences in computational methodologies, toxin complexity, and validation strategies. The systematic bias assessment conducted in this review underscores the critical need for standardized frameworks to guide epitope prediction and validation efforts. Key areas requiring attention include the careful selection of training datasets, optimization, and transparent reporting of algorithm parameters, and the adoption of rigorous, multitechnique validation protocols. Furthermore, the field must urgently address the scarcity of curated venom-specific datasets and establish standardized benchmarks to improve reproducibility and cross-study comparability. Overall, immunoinformatics-driven discovery offers a transformative pathway for venom research and antivenom development. However, realizing its full potential will depend on collaborative efforts to enhance data quality, refine computational models, and integrate experimental validation within standardized methodological frameworks.

**Key Points**

- Studies employing multitool computational approaches showed higher rates of successful epitope confirmation compared to those using single prediction models.
- The lack of venom-specific data in training sets of AI-based tools limits prediction accuracy and increases the risk of false positives.
- Major challenges include methodological heterogeneity, underreporting of negative results, and the absence of standardized frameworks for bias assessment.
- Integrating diverse computational tools with standardized experimental validation is essential for advancing immunoinformatics-driven antivenom development.

## Supplementary Material

Figure_S3_bbaf519

Table_S1_bbaf519

Table_S2_bbaf519

## Data Availability

All relevant data are within the paper and its Supporting information files.

## First author biography

Razana Zegrari is a PhD student focusing on computational immunology and antivenom research, currently enrolled at Mohammed VI University of Sciences and Health, Casablanca, Morocco.
