# Supplementary figures and images for: Computational immunology in venom research: a systematic review of epitope prediction and validation approaches
Source: Brief Bioinform. 2025 Oct 3;26(5):bbaf519. doi: 10.1093/bib/bbaf519 (PMC12494218; doi:10.1093/bib/bbaf519)

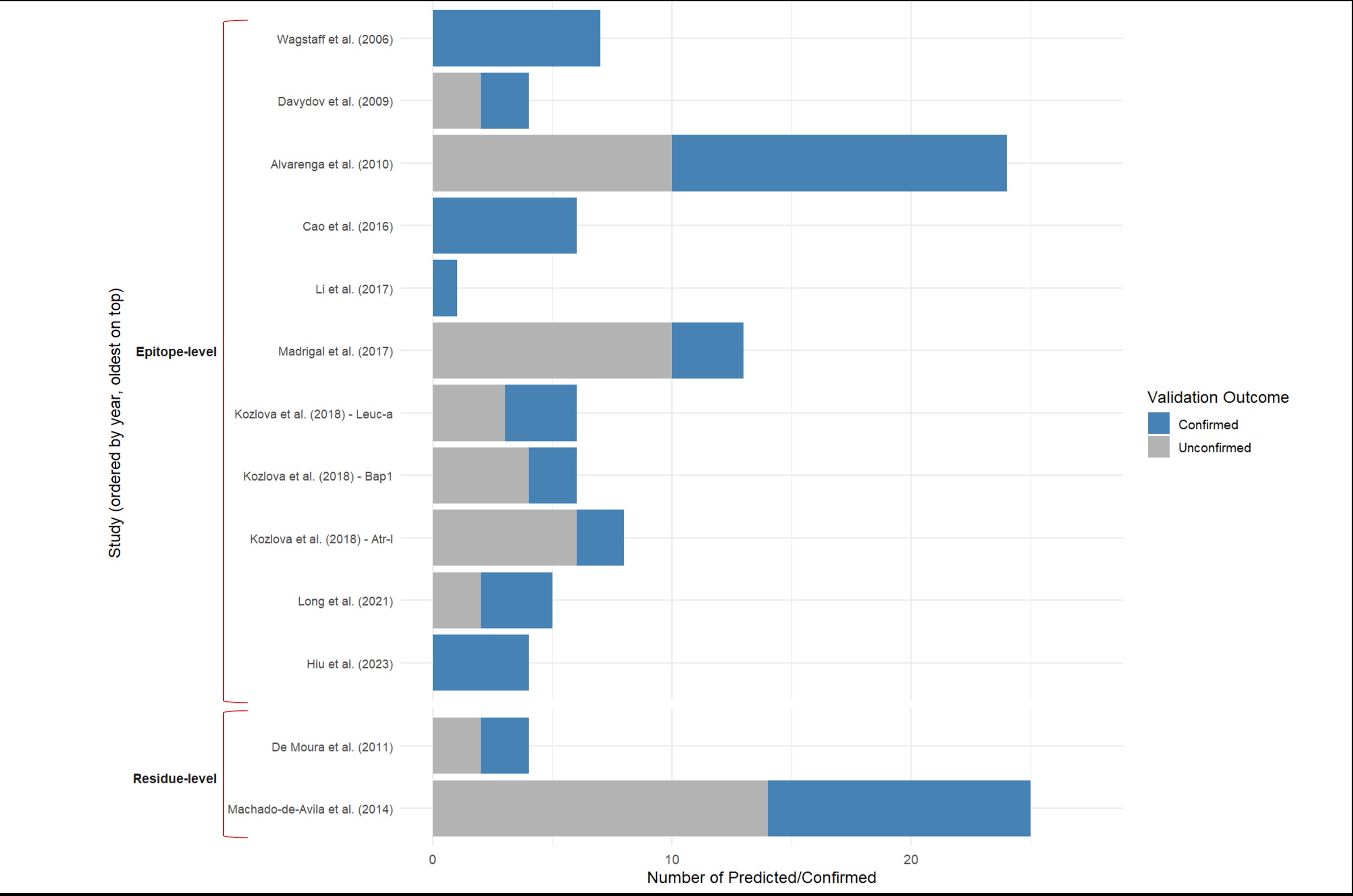

Supplement: Figure_S3_bbaf519 [file figure_s3_bbaf519.jpeg]
