# Supplementary material for: Computational immunology in venom research: a systematic review of epitope prediction and validation approaches
Source: Brief Bioinform. 2025 Oct 3;26(5):bbaf519. doi: 10.1093/bib/bbaf519 (PMC12494218; doi:10.1093/bib/bbaf519)
Supplement: Table_S1_bbaf519 [file table_s1_bbaf519.docx]

| **Number**  **Supplementary Table S1. Screening and Eligibility Outcomes with Rationale for Study Exclusion** | **Title** | **Argument for exclusion** |
| --- | --- | --- |
| 1 | Virtual prediction of potential immunogenic epitope of candoxin protein from Malayan krait (Bungarus candidus) venom | Computational predictions only, no experimental validation |
| 2 | Antigenic epitopes prediction and MHC binder of a paralytic insecticidal toxin (ITX-1) of Tegenaria agrestis (hobo spider) | Computational predictions only, no experimental validation |
| 3 | Prediction of epitopes in closely related proteins using a new algorithm | SELECTED (epitope predicted in silico and experimentally validated) |
| 4 | Identification of B cell recognized linear epitopes in a snake venom serine proteinase from the central American bushmaster Lachesis stenophrys | SELECTED (epitope predicted in silico and experimentally validated) |
| 5 | Immunogenicity of snake α-neurotoxins and the CD4 T cell epitopes | Computational predictions only, no experimental validation |
| 6 | Protection against the toxic effects of Loxosceles intermedia spider venom elicited by mimotope peptides | SELECTED (epitope predicted in silico and experimentally validated) |
| 7 | An Immunoinformatics Approach to Design Synthetic Peptide Vaccine from Dendroaspis polylepis polylepis Dendrotoxin-K (DTX-K) | Computational predictions only, no experimental validation |
| 8 | Induction of neutralizing antibodies against mutalysin-II from Lachesis muta muta Snake Venom Elicited by a conformational B-cell epitope predicted by Blue Star Sting data base. | SELECTED (epitope predicted in silico and experimentally validated) |
| 9 | A Strategy for Efficient Preparation of Genus-Specific Diagnostic Antibodies for Snakebites | SELECTED (epitope predicted in silico and experimentally validated) |
| 10 | Design of antibody-reactive peptides from discontinuous parts of scorpion toxins | SELECTED (epitope predicted in silico and experimentally validated) |
| 11 | Computer-Aided Analysis of West Sub-Saharan Africa Snakes Venom towards the Design of Epitope-Based Poly-Specific Antivenoms | Computational pipeline only, no experimental validation |
| 12 | Unveiling the functional epitopes of cobra venom cytotoxin by immunoinformatics and epitope-omic analyses | SELECTED (epitope predicted in silico and experimentally validated) |
| 13 | An immunoinformatic approach to assessing the immunogenic capacity of alpha-neurotoxins in elapid snake venoms | Immunoinformatic predictions only, no experimental validation |
| 14 | Preparation of monoclonal antibodies against gamma-type phospholipase A2 inhibitors and immunodetection of these proteins in snake blood | SELECTED (epitope predicted in silico and experimentally validated) |
| 15 | Computational B-cell epitope identification and production of neutralizing murine antibodies against Atroxlysin-I | SELECTED (epitope predicted in silico and experimentally validated) |
| 16 | Design of antibody-reactive peptides from discontinuous parts of scorpion toxins | SELECTED (epitope predicted in silico and experimentally validated) |
| 17 | Prediction of an epitope-based computational vaccine strategy for gaining concurrent immunization against the venom proteins of Australian Box Jellyfish | Computational predictions and docking only, no experimental validation |
| 18 | An immunoinformatics approach toward epitope-based vaccine design through computational tools from Bungarus caeruleus's neurotoxin | Immunoinformatic predictions and docking only, no experimental validation |
| 19 | B and t-cell epitopes based vaccine design in api m3 allergen of apis mellifera: An immunoinformatics approach | Immunoprediction of epitopes for allergens from bee venom with no experimental validation |
| 20 | Bioinformatics-based design of novel antigenic B-cell linear epitopes of Deinagkistrodon acutus venom | SELECTED (epitope predicted in silico and experimentally validated) |
| 21 | Potential and limitations of epitope mapping and molecular targeting in Hymenoptera venom allergy | Review |
| 22 | Discontinuous epitope prediction based on mimotope analysis | Methodological paper describing a computational tool (MIMOP), no original experimental validation |
| 23 | Recombinant vaccine design against Clostridium spp. toxins using immunoinformatics tools | Book chapter/review |
| 24 | Structural analysis and antigenic epitope prediction of snake venom C type lectin family proteins. | Computational antigenic epitope prediction only no experimental validation |
| 25 | Equatorial Spitting Cobra (Naja sumatrana) from Malaysia (Negeri Sembilan and Penang), Southern Thailand, and Sumatra : Comparative Venom Proteomics, Immunoreactivity and Cross-Neutralization by Antivenom | Experimental proteomic and antivenom neutralization study, no computational epitope prediction |
| 26 | Snake Venomics : Fundamentals, Recent Updates, and a Look to the Next Decade | Review |
| 27 | Recombinant Protein Containing B-Cell Epitopes of Different Loxosceles Spider Toxins Generates Neutralizing Antibodies in Immunized Rabbits | Wrong study design : epitopes were identified using experimental SPOT-synthesis and not computational prediction |
| 28 | Innovative Immunization Strategies for Antivenom Development | Review |
| 30 | Anti-Metalloprotease P-I Single-Domain Antibodies : Tools for Next-Generation Snakebite Antivenoms | Wrong study design: antibodies were generated from experimental immunization with purified toxin; in silico docking was used only as a complementary analysis, not as the starting method for epitope prediction |
| 31 | Prediction of major histocompatibility complex binding peptides and epitopes from Naja naja Cardiotoxin (CTX) | Computational MHC-binding predictions only, no experimental validation |
| 32 | An immunoinformatic approach to assessing the immunogenic capacity of alpha-neurotoxins in elapid snake venoms | Immunoinformatic predictions only, no experimental validation |
| 33 | Molecular, immunological, and biological characterization of Tityus serrulatus venom hyaluronidase: new insights into its role in envenomation | Wrong study design: epitope mapping was carried out experimentally (SPOT-synthesis, antibody binding) |
| 34 | Neutralization of hemorrhagic snake venom metalloproteinase HR1a from Protobothrops flavoviridis by human monoclonal antibody | Wrong study design: epitope mapping was performed experimentally with overlapping peptides and confirmed by modeling |
| 35 | In silico rational design of a novel tetra-epitope tetanus vaccine with complete population coverage using developed immunoinformatics and surface epitope mapping approaches | Computational vaccine design only, no experimental validation |
| 36 | Synthetic peptides to produce antivenoms against the Cys-rich toxins of arachnids | Review |
